# Potential of Gold Nanoparticles in Current Radiotherapy Using a Co-Culture Model of Cancer Cells and Cancer Associated Fibroblasts Cells

**DOI:** 10.3390/cancers14153586

**Published:** 2022-07-22

**Authors:** Abdulaziz Alhussan, Nicholas Palmerley, Julian Smazynski, Joanna Karasinska, Daniel J. Renouf, David F. Schaeffer, Wayne Beckham, Abraham S. Alexander, Devika B. Chithrani

**Affiliations:** 1 Department of Physics and Astronomy, University of Victoria, Victoria, BC V8P 5C2, Canada; alhussan@uvic.ca (A.A.); nicholaspalmerley@uvic.ca (N.P.); wbeckham@bccancer.bc.ca (W.B.); 2 Deeley Research Centre, British Columbia Cancer—Victoria, Victoria, BC V8R 6V5, Canada; jsmazynski@bccrc.ca; 3 Pancreas Centre BC, Vancouver, BC V5Z 1G1, Canada; jkarasinska@bccrc.ca (J.K.); drenouf@bccancer.bc.ca (D.J.R.); david.schaeffer@vch.ca (D.F.S.); 4 Department of Pathology & Laboratory Medicine, University of British Columbia, Vancouver, BC V6T 1Z7, Canada; 5 Radiation Oncology, British Columbia Cancer—Victoria, Victoria, BC V8R 6V5, Canada; aalexander3@bccancer.bc.ca; 6 Centre for Advanced Materials and Related Technologies, Department of Chemistry, University of Victoria, Victoria, BC V8P 5C2, Canada; 7 Centre for Biomedical Research, Department of Biology, University of Victoria, Victoria, BC V8P 5C2, Canada; 8 Department of Medical Sciences, University of Victoria, Victoria, BC V8P 5C2, Canada; 9 Department of Computer Science, Mathematics, Physics and Statistics, Okanagan Campus, University of British Columbia, Kelowna, BC V1V 1V7, Canada

**Keywords:** MIA PaCa-2, cancer associated fibroblasts, co-culture, monoculture, gold nanoparticle, pancreatic cancer, nanotechnology, radiosensitization

## Abstract

**Simple Summary:**

Many cancer therapeutics do not account for the complexity of the tumor microenvironment (TME), which may result in failure when applied clinically. In this paper we utilized a simple tumor model made of two types of pancreatic cancer cells that contribute to the tumor environment, i.e., cancer cells and cancer associated fibroblasts. Herein, radiotherapy along with radiosensitizing gold nanoparticles were used to test the efficacy of a co-culture vs. monoculture model. The results show that the co-culture model exhibited heightened resistance to radiation. Furthermore, we found that the combination of gold radiosensitizers with radiotherapy reduced the radioresistance of the co-culture model compared to radiotherapy alone. This study demonstrates the potential of using nanotherapeutics in targeting the complex tumor microenvironment.

**Abstract:**

Many cancer therapeutics are tested in vitro using only tumour cells. However, the tumour promoting effect of cancer associated fibroblasts (CAFs) within the tumour microenvironment (TME) is thought to reduce cancer therapeutics’ efficacy. We have chosen pancreatic ductal adenocarcinoma (PDAC) as our tumor model. Our goal is to create a co-culture of CAFs and tumour cells to model the interaction between cancer and stromal cells in the TME and allow for better testing of therapeutic combinations. To test the proposed co-culture model, a gold nanoparticle (GNP) mediated-radiation response was used. Cells were grown in co-culture with different ratios of CAFs to cancer cells. MIA PaCa-2 was used as our PDAC cancer cell line. Co-cultured cells were treated with 2 Gy of radiation following GNP incubation. DNA damage and cell proliferation were examined to assess the combined effect of radiation and GNPs. Cancer cells in co-culture exhibited up to a 23% decrease in DNA double strand breaks (DSB) and up to a 35% increase in proliferation compared to monocultures. GNP/Radiotherapy (RT) induced up to a 25% increase in DNA DSBs and up to a 15% decrease in proliferation compared to RT alone in both monocultured and co-cultured cells. The observed resistance in the co-culture system may be attributed to the role of CAFs in supporting cancer cells. Moreover, we were able to reduce the activity of CAFs using GNPs during radiation treatment. Indeed, CAFs internalize a significantly higher number of GNPs, which may have led to the reduction in their activity. One reason experimental therapeutics fail in clinical trials relates to limitations in the pre-clinical models that lack a true representation of the TME. We have demonstrated a co-culture platform to test GNP/RT in a clinically relevant environment.

## 1. Introduction

**Figure 1:**
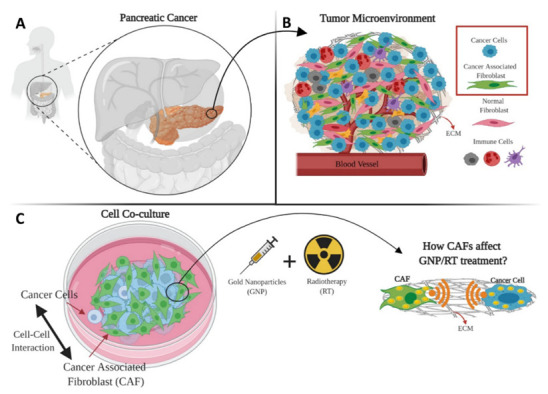
Schematic showing the complexity of the pancreatic cancer tumour microenvironment (TME) and possible targeting using gold nanoparticles (GNPs) as radiosensitizers. (**A**) Pancreatic cancer location in the proximity of many vital organs. (**B**) Two of the most important components of the TME are cancer cells and cancer-associated fibroblasts (CAFs) which coexist in co-culture. (**C**) Cancer cells and CAFs were grown together in co-culture for cell-to-cell interaction to occur.

Pancreatic ductal adenocarcinoma (PDAC) has the highest mortality rate of all major cancers, with a 5-year mortality rate higher than 92% and most patients succumbing to their disease within the first year [1,2]. It is the third leading cause of cancer deaths in both the US and in Canada according to the National Cancer Institute and the Canadian Cancer Society, respectively. At present, surgical resection followed by adjuvant chemotherapy is the most favorable therapy for early stage pancreatic cancer [3]. Nevertheless, pancreatic cancer is locally invasive and highly metastatic, rendering most patients not suitable for surgery. Alternatively, radiotherapy (RT) has limitations in safely targeting pancreatic cancer. The pancreas is located in the vicinity of multiple organs at risk for radiotherapy-associated toxicity (Figure 1A), which limits the desired radiation dose needed for local control [4,5]. Moreover, the existence of the tumour microenvironment (TME), which contains a heterogenous mix of cancer associated fibroblasts (CAFs), immune cells, macrophages, vasculatures, and the extracellular matrix (ECM), inhibits the spread of chemotherapeutic drugs to the tumour (Figure 1B) [6,7]. Not only do CAFs encompass up to 85% of the overall stromal cell population, but they also promote tumour growth, angiogenesis, metastasis, and resistance to chemotherapy and radiotherapy [3,8,9,10,11]. The ECM in pancreatic cancers produce a very dense fibrotic stroma that has a high percentage of CAF activation, which decreases blood vessel formation, limiting drug delivery and leading to tissue hypoxia, thus reducing the effectiveness of RT [12]. When activated, CAFs help with the deposition of the ECM which supports the development and expansion of fibrotic PDAC stroma and accounts for the majority of the tumour volume [13]. Cancer cells and CAFs often work in tandem, wherein cancer cells stimulate the activation of CAFs to encourage the growth of the tumour, while CAFs secrete numerous growth factors such as hepatocyte growth factor (HGF) and transforming growth factor beta (TGF-β), and by secretion of host-derived cytokines and chemokines that drive the tumour to develop and disseminate [14,15].

This intercommunication between cancer cells and CAFs fosters the ideal niche for tumours to develop. Therefore, developing new technologies that target both tumour cells and CAFs warrants further investigation. Nanoparticles (NPs) of high-Z materials such as gold nanoparticles (GNPs) have shown promising results as radiosensitizing agents in radiotherapy, and as vectors for targeted-drug delivery in chemotherapy [4,5,16]. GNPs are particularly promising due to their simple surface chemistry, biocompatibility, low toxicity, and ease of manufacturing [17]. RT was chosen as the therapeutic intervention of the co-culture model compared to monoculture because it is a standard local treatment option for unresectable disease. Furthermore, GNPs were added to the RT protocol since GNPs are successful radiosensitizing agents. There is evidence that GNPs and low concentrations of anticancer drugs can sensitize cancer cells to radiotherapy in an in vitro monoculture environment [18,19,20,21,22]. GNPs enter cells primarily via receptor-mediated endocytosis (RME) [23,24,25,26]. When GNPs are introduced to RT they act as radiosensitizers. This radiosensitization is mediated by the photoelectric absorption/effect [27]. This interaction occurs when an incident photon interacts with an inner-shell electron, transferring all its energy to it and causing it to be ejected from the atom as secondary electrons. Outer-shell electrons can fill this vacancy, liberating further energy often in the form of additional secondary Auger electrons. The absorption coefficient for the photoelectric effect scales roughly with 𝑍 ^3^/𝐸^3^, where Z is the atomic number and E is the energy of the incident photon. Therefore, materials like gold (𝑍 = 79) can have a much higher absorption than tissue (𝑍~7.5) [28]. The dose enhancement and radiosensitization effect is attributed to the increased number of photoelectric absorption events leading to the production of low-energy secondary electrons scattering from the surface of the high-Z material [29,30]. The resulting inner shell vacancy can initiate further secondary electrons in the form of an Auger cascade, spreading out much of the remaining energy of the interaction into more low-energy electrons [31]. The net effect is a spray of short-range electrons that can cause many ionizations as they slow in the surrounding medium. These electrons can directly interact with the DNA causing DSBs. Alternatively, they can act indirectly, generating free radicals which then promote DNA DSBs, amplifying cell death [32,33]. 

The uptake of GNPs in co-culture models of pancreatic tumours using different ratios of CAFs to tumour cells and subsequent radiation treatment has not been investigated previously based on the literature. Understanding the effect of the interactions between different cell lines in co-culture systems on various treatment modalities is critical to reap the benefits of nanotechnology, especially for pancreatic cancer. Insight into the interactions of GNPs with tumour cells and CAFs in co-culture systems will help inform more in-depth co-culture experiments before transitioning to 3D in vitro co-culture systems and then to in vivo models. This paper sheds light on the behavior and the effect of GNPs in a co-culture of pancreatic cancer cells and CAFs compared to monoculture, and how different ratios of CAFs affect the overall treatment of cancer cells (Figure 1C). CAFs and cancer cells were grown together in co-cultures at different ratios to mimic the real-life variations in the TME [3,11]. A co-culture model is developed to test the difference in outcome of GNP-mediated radiation response in a more clinically relevant co-culture environment. We aim to address three main questions:

1. What effect do CAFs have on cancer cells in a co-culture environment in relation to GNP uptake, proliferation, and DNA damage?
2. How advantageous is the GNPs/RT treatment vs. RT alone in monoculture vs. co-culture?
3. Does the ratio of CAFs to cancer cells affect GNP uptake, DNA DSB, and the proliferation of cells?

## 2. Materials and Methods

### 2.1. Gold Nanoparticle Synthesis, Functionalization and Characterization

A citrate reduction method was used to produce gold nanoparticles (GNPs) of 13.2 ± 1.3 nm diameter in size. To synthesize GNPs, 900 µL of 1% Tetrachloroauric (III) acid trihydrate (AuCl_4_H·3H_2_O) gold salt solution was added to 90 mL of double-distilled water in an Erlenmeyer flask, stirred and boiled. As soon as it started to boil, 1800 µL of the 1% sodium citrate tribasic dihydrate (HOC(COONa)(CH_2_COONa)_2_·2H_2_O) reducing agent was added and stirred while boiling for 10 min. The solution colour changed to red because of the formation of GNPs. The heat was then turned off, and the solution was stirred at room temperature for 10 more minutes. Polyethylene glycol (PEG) and RGD peptides were used at a surface density of 1 PEG per nm^2^ of the GNP surface area and at one molecule of RGD for every two PEG molecules to functionalize the negatively charged GNPs to create GNP_PEG-RGD_. The former was added to simulate an in vivo environment in which PEG would help nanoparticles avoid the immune system, and the latter was added to improve the nanoparticles’ uptake into cancer cells. 

For live cell confocal imaging, RGD peptides and PEG-thiol-CY5 were used to create GNP_PEG–CY5–RGD_ (fluorescent CY5 dye; ~651 nm excitation, ~670 nm emission). For flow cytometry analysis, GNP_PEG–FITC–RGD_ (fluorescent FITC dye; ~490 nm excitation, ~525 nm emission) were used. A Perkin Elmer λ 365 ultraviolet visible (UV-VIS) spectrophotometer, and ζ potential were used to determine the size, concentration, and surface charge of GNPs, GNP_PEG_, and GNP_PEG–RGD_, respectively and verify the conjugation of PEG and RGD to the nanoparticles. The GNPs shape and size were verified using Transmission Electron Microscopy (TEM) (Ultra-high Resolution Scanning Electron Microscope SU9000, Hitachi, Pleasanton, CA, USA).

### 2.2. Cell Culture Methodology

The human pancreatic cancer cell line MIA PaCa-2 (ATCC#: CRL-1420™) was obtained from the American Type Culture Collection. Human pancreatic cancer-associated fibroblasts (CAF-98) were derived from resected PDAC tumour tissue from a consenting patient through the Gastrointestinal (GI) Biobank at the Vancouver General Hospital. All cells were cultured in high glucose Dulbecco’s Modified Eagle’s Medium (DMEM; Gibco, ThermoFisher Scientific, Waltham, MA, USA) enriched with 10% fetal bovine serum (FBS; Gibco), 1% penicillin/streptomycin (Gibco), and 4 mM of GlutaMax (Gibco). For cell detachment and cell fixations, trypsin–EDTA(Gibco) and paraformaldehyde (PFA; Sigma Aldrich, Oakville, ON, Canada) were used, respectively. Phosphate-buffered saline (PBS) was used for cell washing, and cell incubations occurred at 37 °C with 5% CO_2_. Cells were seeded at three different ratios of CAF98 to MIA PaCa-2, 2:1, 5:1, and 10:1, and then they were incubated for three days.

### 2.3. Image Preparation

Confocal microscopy (Zeiss LSM 980,Carl Zeiss Microscopy GmbH, Jena, Germany) was used to visualize GNP distribution in cells. Live cells were imaged using an oil-immersion 60X lens. Both monoculture and co-culture cells were cultured on 35 mm coverslip-bottom dishes (MatTek, Ashland, MA, USA) with 2 mL of media and incubated for 72 h. All cells were dosed with 7.5 µg/mL of GNPPEG-CY5-RGD post-incubation for 24 h. Prior to imaging, the media was substituted with colourless media (FluoroBrite DMEM; Gibco, ThermoFisher Scientific, Waltham, MA, USA) and four drops of NucBlue^®^, ThermoFisher Scientific, Waltham, MA, USA) Live reagent (Hoechst^®^ 33,342 dye; ~350 nm excitation, ~461 nm emission, ThermoFisher Scientific, Waltham, MA, USA) was added to stain the nucleus of each cell. These samples were then incubated for 20 min before imaging. 

Darkfield (DF) coupled with hyperspectral imaging (HSI) CytoViva microscope (CytoViva, Auburn, AL, USA) were used to determine GNP localization within cells. Fixed cells were imaged for DF and HSI under a 60× objective. For DF and HSI, cells were grown on coverslips at the bottom of 6-well dishes with 3 mL of media and were incubated for 72 h. Cells were then dosed with 7.5 µg/mL of GNPPEG−RGD and were incubated for 24 h. Cells were then washed three times with 1 mL of PBS followed by adding 1 mL of 4% paraformaldehyde for fixation and incubated at 37 °C with 5% CO_2_. After a 20 min incubation period, cover slips were rinsed three times with PBS, removed from their wells, and mounted to a glass slide using Permount mounting medium (Fisher Scientific Company, Ottawa, ON, Canada).

### 2.4. Flow Cytometry and Magnetic Bead Isolation

MIA PaCa-2 and CAF98 cell lines were cultured either independently or in co-culture and labelled with FITC conjugated gold nanoparticles. Labelled cell lines were run on a Cytek Aurora spectral flow cytometer (Cytek, Fremont, CA, USA) to measure percent expression and median fluorescent intensity of the FITC signal. To determine the proportion of CAF98 fibroblast cells within the co-culture, cells were first blocked with Anti-Hu Fc receptor block (Biolegend, Cat: 422302) then stained in FACS buffer (PBS + 2% FBS) plus anti-Fibroblast PE antibody (Miltenyi, Cat: 130-126-007) to stain CAF98 fibroblast cells. Data was unmixed and manually gated using SpectroFlo Software (Cytek, Fremont, CA, USA) to determine percent expression and median fluorescent intensity. To isolate CAF98 cells from the co-cultured MIA PaCa-2 cells, a bead enrichment kit, Anti-Fibroblast MicroBeads, human (Cat #: 130-050-601), was used to separate human fibroblasts. This method involves targeting cells using antibodies or ligands directed against specific cell surface antigens. CAFs are labelled with antibodies and magnetic particles that can be immobilized once an electromagnetic field is applied. Both CAFs and cancer cells can be easily separated and recovered for further processing by sending them through a magnetic lens. Enrichment was verified by the same fibroblast staining method described above.

### 2.5. Immunofluorescence Assay

The extent of DNA DSBs damage is examined using an optically labelled antibody against the repair protein, γ-H2AX. A primary antibody and an optically labelled secondary antibody are used for the assay. Cells are incubated on glass coverslips in 6-well dishes. 24-h post treatment, cells are washed with PBS, fixed with 4% PFA for 5 min, then washed again with PBS, and treated with 2% BSA/0.1% Triton-X in PBS for 20 min to reduce background noise. The γ-H2AX primary antibody is diluted 1:200 in 0.5% BSA/0.1% Triton-X/PBS, while the secondary antibody was diluted 1:500 in 0.5% BSA/0.1% Triton-X/PBS. The coverslips are first incubated with the primary antibody, followed by washing with PBS. Cells are then rinsed with 0.5% BSA/0.175% Tween-20/PBS for 5-min and are incubated with secondary antibody in the dark for 30 min. Following the incubation, cells are rinsed in PBS, dried; and then mounted to glass coverslips with ProLong™ Glass Antifade Mountant for imaging.

### 2.6. Proliferation Assay

This assay is conducted to determine the cytotoxicity of certain stimulants (e.g., radiation or chemotherapeutic drugs) on cells. About 10^4^ of the treated cells are seeded in each well of black-walled clear-bottom 96-well plates (Greiner) with 100 μL fresh media, leaving one column empty for control, and covered with a breathable membrane to reduce evaporation (Breathe-Easier Membranes). After 24 h, the membrane is disposed of, the media is removed, and the samples are washed with PBS and then are incubated for an additional period of 48 h in fresh media. After 48 h, the media is substituted with media containing 10% *v/v* resazurin dye (PrestoBlue, Thermo-Fisher, Waltham, MA, USA) followed by incubation for 30 min. Plates are then mixed for 5 min, and luminescence is measured after 25 min incubation. This was repeated for a period of about 8 days, with measurements taken every other day. Fluorescence was measured using a Biotek Cytation 1 plate reader (filters at Excitation 530/25 nm, Emission 590/35 nm).

### 2.7. Statistical Analysis

A statistical analysis was performed using the Welch’s *t*-test via the python package statannot. Where relevant in the figures, * indicates *p* < 0.05, ** indicates *p* < 0.01, *** indicates *p* < 0.001. All experiments were done in triplets (*n* = 3) and the error bars signify one standard deviation from the mean of three independent measurements. 

## 3. Results and Discussion

### 3.1. Gold Nanoparticles Characterization

**Figure 2:**
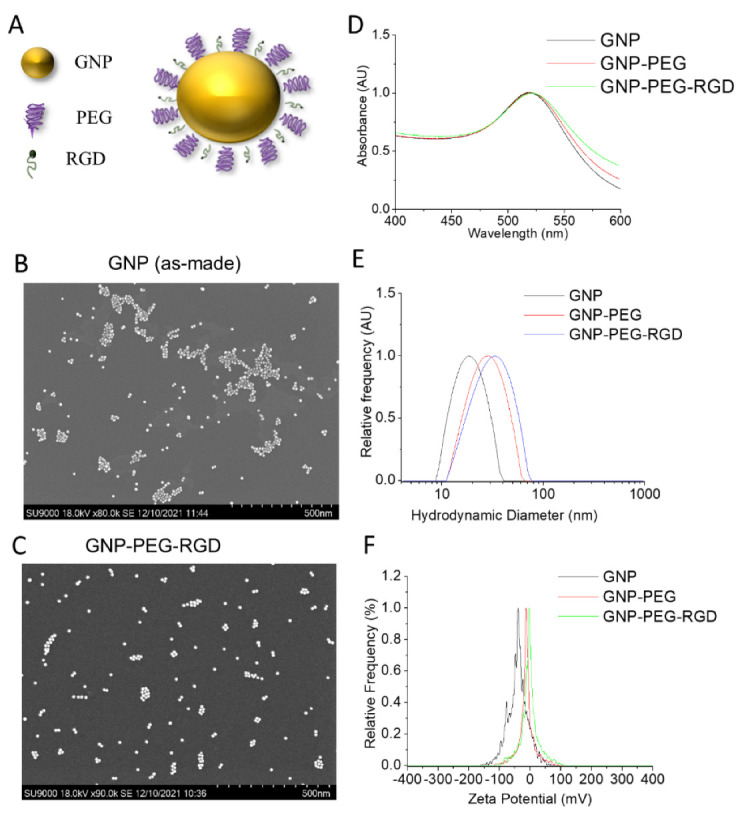
Characterization of the gold nanoparticles (GNPs). (**A**) Schematic showing GNP with measured core diameter of~13.2 functionalized with a polyethylene glycol (PEG) molecule as stabilizing agents, and RGD peptides to improve GNPs uptake. (**B**,**C**) Transmission Electron Microscopy (TEM) Image of as made GNP and GNP_PEG-RGD_. (**D**–**F**) UV-visible absorption spectra, hydrodynamic diameter, and ζ-potential measurements of pure GNPs, GNP_PEG_, and GNP_PEG-RGD_, respectively.

Spherical gold nanoparticles of sizes 10–15 nm demonstrate better penetration and uptake capabilities in 3D tumour models [34]. GNPs were coated with polyethylene glycol (PEG) and RGD peptides at a surface density of 1 PEG per nm^2^ of the GNP surface area and at one molecule of RGD for every two PEG molecules to make GNP_PEG-RGD_, Figure 2A. The former was added to simulate an in vivo environment in which PEG would help nanoparticles avoid the immune system by preventing them from binding to blood plasma proteins, and the latter was added to improve the nanoparticles’ uptake into cancerous cells as cancerous cells overexpress integrins on their surface [26,35,36,37]. The GNPs shape and size were verified using transmission electron microscopy (TEM), Figure 2B,C. Verification was also confirmed using UV–visible absorption spectra, dynamic light scattering (DLS), and ζ-potential measurements of pure GNPs, GNP_PEG_, and GNP_PEG-RGD_, respectively, Figure 2D,F. UV-Vis spectrometry was used to estimate the size and concentration of the GNPs, Figure 2D [23,35]. A small red shift in the peak absorbance was observed when the GNP was coated with PEG and RGD. This is expected, since both PEG and RGD peptides are smaller in size (PEG: 2 k and RGD peptide: 1.7 k) in comparison to the GNPs. However, the surface properties of GNP_PEG-RGD_ have noticeable differences compared to as-made GNPs as found by dynamic light scattering (DLS) and ζ-potential measurements, Figure 2E,F, respectively. As-made GNPs have a hydrodynamic diameter of 19.32 nm with a polydispersity index of 7%, as verified by DLS. On the other hand, GNP_PEG−RGD_ has a diameter of 38.61 nm and a polydispersity index of 14%. The results are expected due to the natural increase of the hydrodynamic diameter with conjugation of molecules on the surface. The ζ-potential was found to be −30.53 mV and 0.18 mV for as-made GNPs and GNP_PEG−RGD_, respectively. As-made GNPs have a negative charge because of the negatively charged citrate molecules on the surface of the NPs. When PEG and RGD are added, citrate molecules get substituted with the neutrally charged PEG molecules and the positively charged RGD peptides, causing a substantial shift in the final charge of the molecule.

### 3.2. Determining the Uptake of Gold Nanoparticles in Co-Culture vs. Monoculture

**Figure 3:**
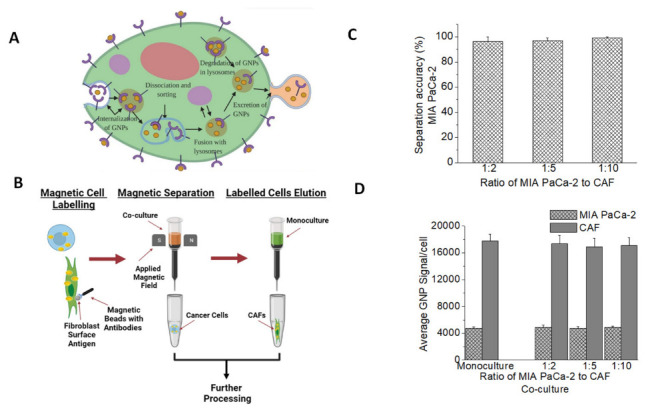
GNP uptake in co-culture vs. monoculture. (**A**) A schematic diagram explains the path of GNPs functionalized for receptor-mediated endocytosis within a cell. (**B**) Magnetic Beads Separation method. The co-culture of MIA PaCa-2and CAFs was stained with fibroblasts surface antigens that attached to CAFs. The co-culture was then washed with magnetic beads with antibodies that link up with the surface fibroblast surface antigen. The cell co-culture tube is then hooked up to a magnet and is washed multiple times, during which the CAFs will be attracted to the magnet and will stay in the tube, whereas MIA PaCa-2 will be collected in a new tube. The CAFs tube is then moved away from the magnetic field and the cells are washed and collected in a new tube. (**C**) Efficiency of the cell separation method. (**D**) Average median fluorescent intensity GNP-FITC signal per cell of MIA PaCa-2and CAFs in monoculture vs. co-culture.

NPs are being effectively used for drug delivery and for tumour imaging in cancer therapy applications. GNPs were chosen as the NP therapeutic agent to test the cancer treatment response of the TME. To develop this co-culture model for our studies, we used CAFs in addition to cancer cells since CAFs are one of the most important cell types that promote tumour growth. The first step in this process is to test how the presence of CAFs affects the GNP uptake of tumour cells. Based on previously published work on monoculture assays, GNPs enter cells predominantly via receptor-mediated endocytosis (Figure 3A) [25,26]. Cell surface receptors attach to the targeting ligands, i.e., RGD on the surface of the GNP, triggering membrane packaging of the GNP with a rise in elastic energy [24,38]. This will allow the GNPs to enter the cells by endocytosis. Receptor-ligand connections restrain the receptor and decrease configuration entropy. Driven by a local reduction in free energy, more receptors diffuse into the encapsulation site, allowing the membrane to completely enclose the particles. When GNP binds to cell surface receptors, membrane invagination occurs, followed by encapsulating the GNPs in endosomes. Internalized GNP is guided within the vesicle and eventually fused to the lysosome for downstream processing. Most receptors are returned to the cell membrane, but processed GNP-containing vesicles go to the cell border for excretion [26,37]. To study the effects of GNP uptake in co-culture vs. monoculture, we have chosen cells from a pancreatic origin: MIA PaCa-2 (cancer cell line) and CAF98 (cancer-associated fibroblast cell line).

Three different ratios of CAF98 to MIA PaCa-2 were chosen to represent heterogeneity in TME. After GNP incubation, cell populations were isolated for quantifying GNP content in each cell line using magnetic beads as outlined in Figure 3B. Co-culture cells were cultured together for a period of 72 h to ensure enough time for intercommunication between cancer cells and CAFs [39]. All cells were dosed with 7.5 µg/mL of GNP_PEG-RGD_ post-incubation for 24 h. This concentration of GNPs is within adequate doses for in vivo and clinical applications [40,41,42,43]. A successful cell isolation or cell sorting would allow us to measure the effects of cell-to-cell interactions on the uptake of nanoparticles in the different cell lines used. The accuracy of the separation method was also tested and over 95% cell separation accuracy was achieved for both cell lines used (Figure 3C). This shows that the collected CAFs post-separation had over 95% CAFs and less than 5% cancer cells, and the collected cancer cells post-separation were ~95% cancer cells and with about 5% CAFs contamination. Images of both cell lines post-separation are shown in Figure S1. To be able to quantify the number of GNPs per cell precisely, we typically use inductively coupled plasma–mass spectrometry (ICP-MS) [44,45,46]. However, due to the inherent risk of using magnetic beads on the mass spectrometer that might lead to damaging the device we opted for using FITC-labelled GNPs which serve the purpose of this study [47]. Labelled cell lines were assessed by flow cytometry to measure percent expression and median fluorescent intensity of the FITC signal in monocultures of MIA PaCa-2 and CAF98, vs. in co-cultures of MIA PaCa-2 and CAF98, Figure 3D, which shows no effect of the co-culture on the GNP uptake of either cell lines. However, the results show that the uptake of GNPs in CAFs is over three times higher than that of cancer cells in both monoculture and co-culture, which is in agreement with our previous experiments that were done in monoculture [21,22]. This is an important observation that might open the door for targeting not only cancer cells but also CAFs using GNPs. The results show that the interaction of tumour cells with CAFs did not significantly affect GNP uptake and retention capacity. 

**Figure 4:**
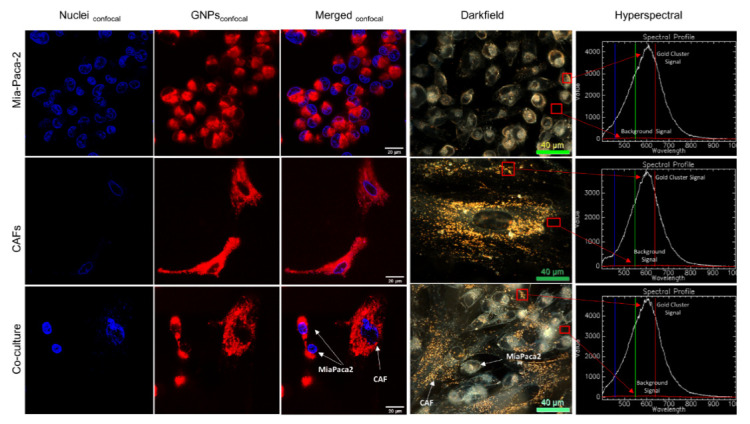
Gold nanoparticles (GNPs) uptake in cancer cells and CAFs in monoculture vs. co-culture. GNP distribution in monoculture MIA PaCa-2 (first row), monoculture CAFs (second row), and co-culture of CAFs & MIA PaCa-2with 2:1 ratio (third row), using confocal imaging in the first three columns, where the first column shows the nucleus in blue, the second column shows GNPs in red, the third column shows both merged, using darkfield microscopy (fourth column), and using hyperspectral imaging (HSI; fifth column) which shows a spectrum collected from GNP clusters (white spectrum) vs. background (red spectrum). Scale bars are 20 μm and 40 μm for confocal and DF, respectively.

Additionally, GNPs distribution in the cells was mapped qualitatively using confocal microscopy and darkfield coupled with hyperspectral imaging (HSI) (see Figure 4). The co-culture images are for a CAF to MiaPaca2 ratio of 2:1. Images of the other two ratios used are available in the Supplementary section (Figure S2). The existence of GNPs in cells was verified using the spectral mapping feature of HSI, as illustrated in the rightmost top corner of the fourth column of Figure 4, where GNP spectra were collected from bright spectra from within cells, confirming that they are from gold from when compared to available data in the imaging library.

### 3.3. Mapping DNA Damage due to GNP-Mediated Radiosensitization in a Co-Culture Model of Cancer Cells and Cancer-Associated Fibroblasts

**Figure 5:**
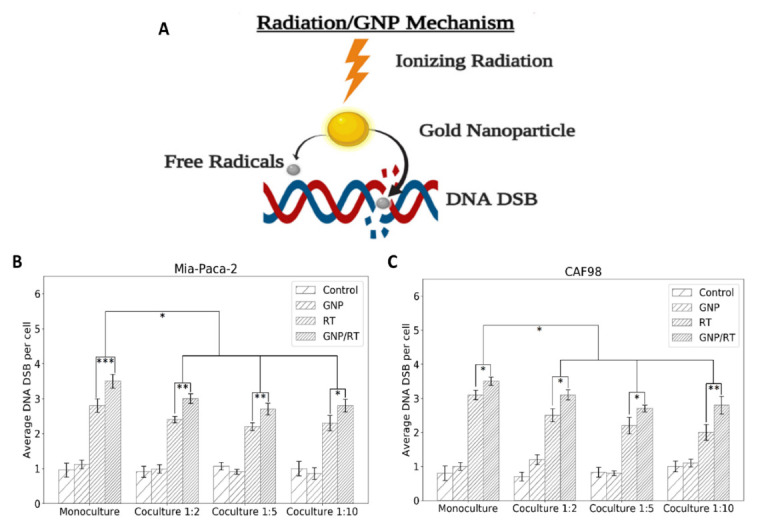
DNA DSB damage in MIA PaCa-2 and in CAFs. (**A**) GNP-mediated radiation damage mechanism. (**B**) Average number of DNA DSB damage per cell in MIA PaCa-2in monoculture vs. in co-culture (*n* = 3). (**C**) Average number of DNA DSB damage per cell in CAFs in monoculture vs. in co-culture (*n* = 3). * indicates *p* < 0.05, ** indicates *p* < 0.01, *** indicates *p* < 0.001.

One of the mechanisms of cell damage due to radiation is through the formation of free radicals that can damage DNA, as explained in the introductory section. Adding GNPs to current radiotherapy (RT) results in an additional number of free radicals causing more DNA damage (Figure 5A), which have been investigated in a monoculture but not in a co-culture [32]. In this study, we investigated how the presence of CAFs affects GNP-mediated DNA damage in cancer cells; we used an immunofluorescence assay to map the DNA damage, and it is typically used as a measure of radiation induced damage [48]. Using this assay, we investigated the effects of radiation and GNPs on co-cultured cells vs. monocultured cells. Specifically, we mapped the DNA double strand breaks (DSBs) since it is the most lethal damage compared to single strand breaks. Antibodies against repair proteins γ-H2AX were used 24-h post radiation treatment of a single dose of 2Gy [49,50]. This allows for the capture of residual damage that typically represents unrepaired DNA DSB damage [51,52].

**Figure 6:**
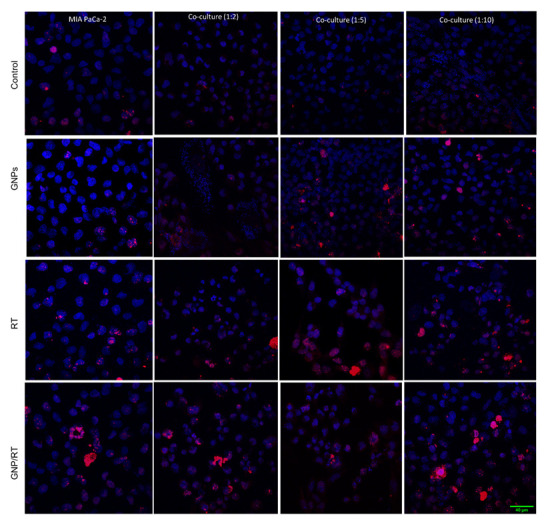
Confocal microscopy images of repair protein γ-H2AX in the nucleus of MIA PaCa-2 and in CAFs. MIA PaCa-2 monoculture (1st column) vs. both MIA PaCa-2 & CAFs co-cultures, 1:2 (2nd column), 1:5 (3rd column), 1:10 (4th column) for control cells (1st row), cells incubated with GNPs (2nd row), irradiated cells (3rd row), irradiated cells with GNP (4th row). Red dots correspond to DNA DSB damages and the blue stains are the cell nuclei. Scale bar is 40 μm.

The average number of foci per cell for MIA PaCa-2 and for CAFs is shown in Figure 5B,C, respectively, where the co-cultures have CAFs to MIA PaCa-2 at ratios of 2:1, 5:1, and 10:1. This was repeated for control cells, cells incubated with GNPs only, irradiated cells only, and cells incubated with GNPs and irradiated. The results lead to three important observations. Firstly, GNPs, in the absence of radiation, did not induce additional DNA DSBs compared to control cells in monoculture or in any of the co-cultures for neither MIA PaCa-2 nor CAFs, which is consistent with our previous experiments, as the amount of GNPs used is tolerated by the cells [53,54]. Secondly, the addition of GNPs to cells prior to radiation induced a statistically significant increase in DNA DSBs compared to irradiated cells without GNPs in monoculture and in all three co-cultures for both MIA PaCa-2 and for CAFs. The increase in the number of foci for GNP/RT cells compared to RT ranges from 22% for MIA PaCa-2 in the 1:10 co-culture to 25% in the monoculture MIA-PaCa-2. The increase in the number of foci for GNP/RT cells compared to RT was 13% for monoculture CAFs and 24%, 23% and 40% for 1:2, 1:5 and 1:10 co-culture, respectively. We attribute this increase in DNA DSB to the radiosensitization and dose enhancement effect of GNPs with RT (Figure 5A), which contributes an increase in photoelectric absorption events that results in short-range electrons in the vicinity of the cell nucleus causing a rise in free radicals that increases DNA DSBs [32,33]. It is important to note that even though CAFs had a much higher amount of gold compared to MIA PaCa-2, their DNA DSBs were not higher than that of MIA-PaCa-2. This may be due to the size of CAFs that allows the GNPs to cluster away from the nuclei, thus decreasing the overall DNA DSB damage expected. Lastly, there is a significant decrease in DNA DSB in both MIA PaCa-2and CAFs that were grown in co-cultures vs. monoculture for both RT only and GNP/RT. The decrease ranges from 14% to 23% for MIA PaCa-2 and from 12% to 35% for CAFs. No significant difference in DNA DSB per cell for MIA PaCa-2 nor for CAFs was observed between the three different co-culture ratios used for RT only nor for GNP/RT. We speculate that this occurs because CAFs are involved in radioresistance by increasing DNA damage repair, as suggested by multiple studies [55,56,57]. Similar acquired radioresistance and improved DNA DSB repair of CAFs of multiple origins including breast, prostate and lung were found by Domogauer et al. [58]. CAFs were found to produce an interferon-related DNA damage resistance gene signature (IRDS) that play an important role in the DNA repair mechanism [57,59,60]. DNA DSB were confirmed visually using confocal microscope images for MIA PaCa-2 in monoculture (first column in Figure 6) and co-culture with different ratios of CAFs (second, third, and fourth columns in Figure 6). The Confocal microscopy images of repair protein γ-H2AX in the nucleus of CAFs isshown in Figure S3. The DNA DSBs data corresponding to the monoculture of CAFs is shown in Figure S4.

### 3.4. Determining the Change in Cell Proliferation Due to GNP-Mediated Radiosensitization in a Co-Culture Model of Cancer Cells and Cancer-Associated Fibroblasts

**Figure 7:**
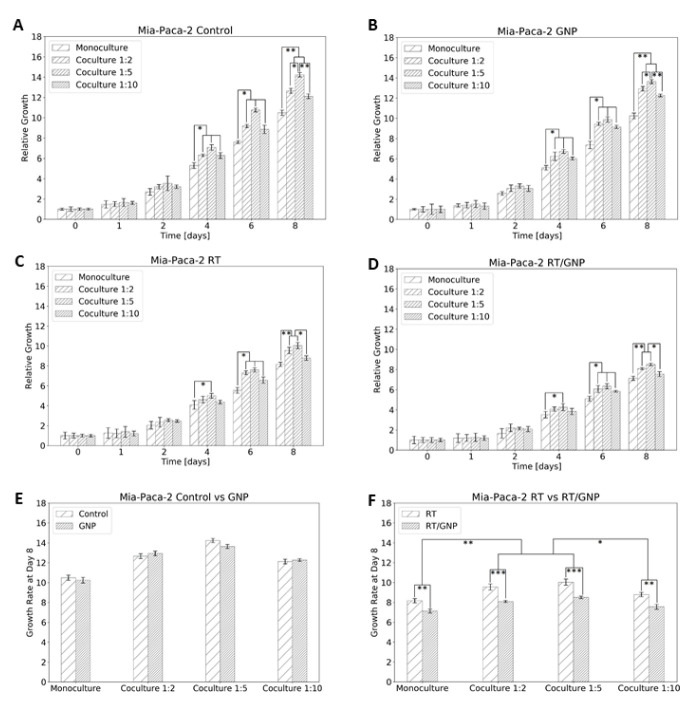
Proliferation assay for Mia-Paca-2. (**A**) Control cells. (**B**) Dosed with GNP. (**C**) Irradiated 2 Gy. (**D**) Dosed with GNP and irradiated 2 Gy. (**E**) Comparison of control vs. GNP at the end of the experiment. (**F**) Comparison of RT vs. RT/GNP at the end of the experiment. * indicates *p* < 0.05, ** indicates *p* < 0.01, *** indicates *p* < 0.001.

Cancer cells divide faster than normal healthy cells, thus most of the therapeutics are targeted towards reducing the proliferation of cancer cells. To determine the effect of the co-culture environment on our proposed therapeutic approach, we used one of the several cell proliferation assays typically conducted to investigate the growth of a cell population over time following a treatment [53,61,62,63,64,65]. Specifically, we used the PrestoBlue assay as we have done previously [53]. PrestoBlue is a resazurin-based dye which measures viable, metabolically active cells via the reduction of resazurin to resorufin and can be detected fluorometrically [65]. The proliferation of cancer cells over time is shown in Figure 7, where the experiment was ended eight days post seeding. The results show a significant increase in cell proliferation for MIA PaCa-2 that was grown in co-cultures with CAFs vs. the MIA PaCa-2 that was grown in monoculture, for all four different conditions, control cells (Figure 7A), cells dosed with GNPs (Figure 7B), irradiated cells (Figure 7C), and irradiated cells dosed with GNPs (Figure 7D). For the control cells, the increase ranges from 16% (for the 1:10 ratio) to 35% (for the 1:5 ratio), for the cells dosed with GNPs it ranges from 20% (for the 1:10) to 33% (for the 1:5), for the irradiated cells it ranges from 8% (for the 1:10) to 23% (for the 1:5), and for the irradiated/GNP dosed cells it ranges from 6% (for the 1:10) to 20% (for the 1:5). The reason behind that is that CAFs not only encourage radioresistance but also promote cancer cell growth through paracrine signals [55,66,67,68,69]. Interestingly, Yang et al. found a substantial decrease in cell proliferation of natural killer cells when co-cultured with irradiated or non-irradiated CAFs vs. when co-cultured with normal fibroblasts [55]. These results reveal the promoting effect of CAFs on cancer cell growth and on the repressing potential of CAFs over noncancerous cells, contributing not only to radioresistance but also to chemoresistance [70]. 

Our results also show no effect of GNPs on the proliferation of cells in either monoculture or co-culture (Figure 7E). However, there was a significant difference in cell proliferation between the three co-cultures used. For example, with the increase of the co-culture ratio from 1:2 to 1:5 there was a significant increase in the proliferation of control MIA PaCa-2 (Figure 7A) and the MIA PaCa-2 dosed with GNPs (Figure 7B), as expected. However, the further increase in the co-culture to 1:10 did not result in an increase in cancer cell proliferation. The reasons are unknown, but we speculate that CAFs are taking over the cell culture and might be starting to starve cancer cells from nutrition. With the introduction of GNPs to the RT, we observed a significant decrease of 13–15% in cancer cell proliferation for the monoculture and the three co-cultures (Figure 7F). This is consistent with the increase of the RT/GNP mediated DNA DSB in cancer cells, which over time leads to more cell deaths and less growth of the tumour (Figure 5 and Figure 6). However, the 1:10 co-culture had significantly lower proliferation compared to the 1:5 and 1:2 co-cultures. The reason is also unknown, but we speculate that it is most likely due to the higher amount of GNP in the co-culture system because of the high rate of internalization in CAFs, therefore affecting the growth of cancer cells when irradiated. 

## 4. Conclusions

Pancreatic cancer remains one of the deadliest cancers. Despite advancements in radiotherapy and chemotherapy, there is an evident need for new therapeutic interventions. At the same time, one of the reasons why many treatments pass in vitro tests but fail clinical trials for pancreatic cancer is the complexity of the pancreatic TME. CAFs are but one vital component of the TME which play an important role in tumour development. In this experiment, we investigated how CAFs affect cancer cells when grown in co-culture, and how the incorporation of GNPs into RT affects the treatment of pancreatic cancer in both monoculture and co-culture. To measure these effects, we measured GNPs uptake, DNA DSB and proliferation assays. In the non-irradiated case, GNPs did not affect cell proliferation or introduce additional DNA DSB in either the monoculture or the co-culture. Co-culture did not affect GNP uptake in either cell lines, even though CAFs had significantly higher GNP uptake compared to cancer cells. A significantly higher proliferation of the cells in co-culture vs. monoculture was found, suggesting that CAFs promote tumour cell growth. Furthermore, for irradiated cells, the co-culture significantly decreased DNA DSBs compared to the monoculture. No significant difference was found between the different co-cultures in the DNA DSB assay. However, the proliferation assay shows a significant difference between the three co-cultures used. The results show evidence of resistance to radiation when cancer cells are grown in co-culture with CAFs. Furthermore, the incorporation of GNPs in RT caused a significant radiosensitization effect compared to RT alone in both monoculture and co-cultures of cancer cells and of CAFs. This opens the door for new treatment modalities involving GNPs as a radiosensitizing agent to target both cell lines. Further studies are needed to study the exact mechanism of CAFs radioresistance in co-culture. Overall, the results indicate the importance of targeting both cancer cells and CAFs. The development of new treatments that could silence CAFs may be an important development towards improving the therapeutic options for pancreatic cancer. 

## Data Availability

Datasets generated and/or analyzed during the current study are available from the corresponding author on reasonable request.

## Supplementary Materials

The following are available online at https://www.mdpi.com/article/10.3390/cancers14153586/s1, Figure S1: (**A**) Tables showing the percentage of Mia-Paca-2 and CAFs post-separation in three used ratios for this experiment. (**B**) Phase contrast microscope of Mia-Paca-2 and CAFs post-separation; Figure S2: Darkfield Images of Gold nanoparticle Uptake. Top: using a ratio of 5:1 CAFs to cancer cells. Bottom: using 10:1 ratio of CAFs to cancer cells. Scale bar: 40 μm; Figure S3: Confocal microscopy images of repair protein γ-H2AX in the nucleus of CAFs. Left: irradiated cells. Right: irradiated cells with GNP. Red dots correspond to DNA DSB damages and the blue stains are the cell nuclei. Figure S4: Mia-Paca-2 proliferation RT vs. RT/GNP at the end of the experiment for monoculture vs. the 3 different co-cultures.
