# Supplementary material for: Potential of Gold Nanoparticles in Current Radiotherapy Using a Co-Culture Model of Cancer Cells and Cancer Associated Fibroblasts Cells
Source: Cancers (Basel). 2022 Jul 22;14(15):3586. doi: 10.3390/cancers14153586 (PMC9332249; doi:10.3390/cancers14153586)
Supplement: Supplementary file 1 [file cancers-14-03586-s001.zip › cancers-1790659-supplementary.pdf]

A)

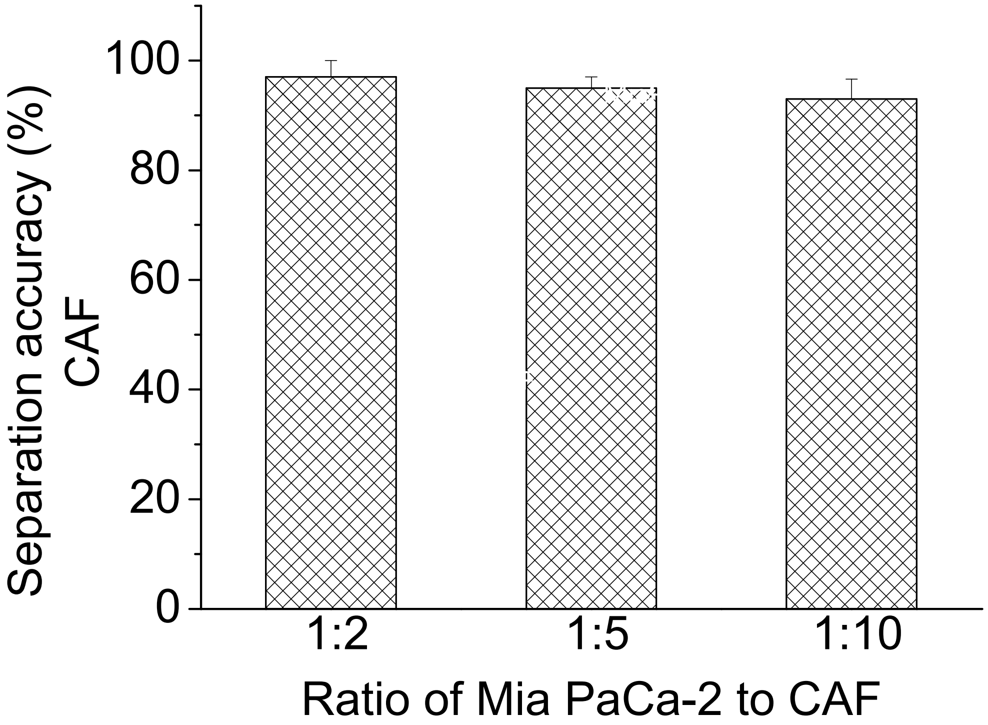

B)

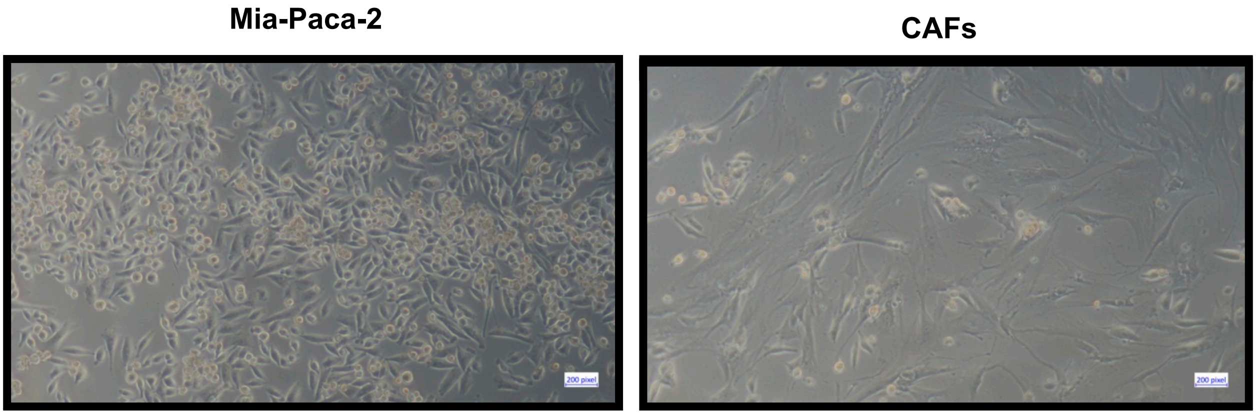

Figure S1. A) Tables showing the percentage of Mia-Paca-2 and CAFs post-separation in three used ratios for this experiment**. B)** Phase contrast microscope of Mia-Paca-2 and CAFs post-separation.

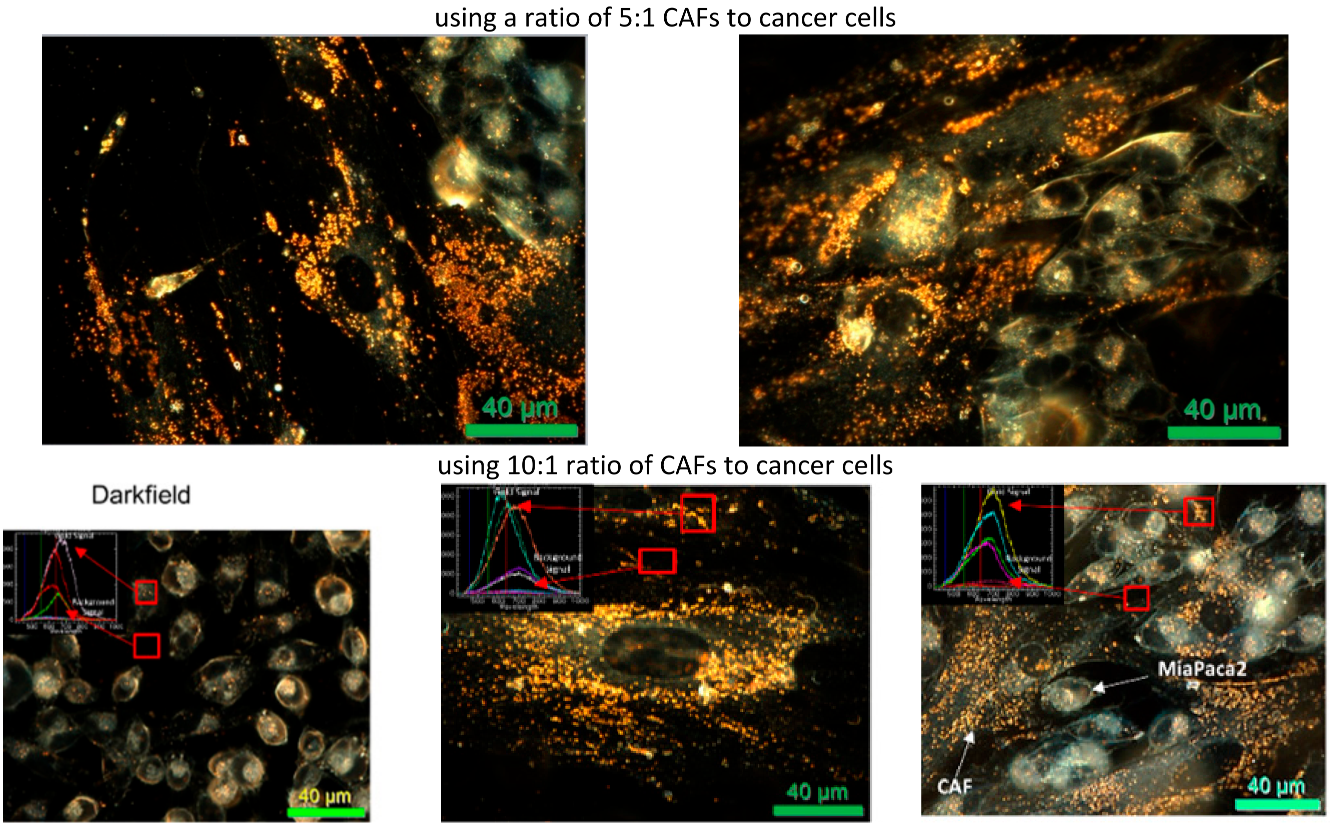

Figure S2. 1. Darkfield Images of Gold nanoparticle Uptake. Top: using a ratio of 5:1 CAFs to cancer cells. Bottom: using 10:1 ratio of CAFs to cancer cells. Scale bar: 40 μm.

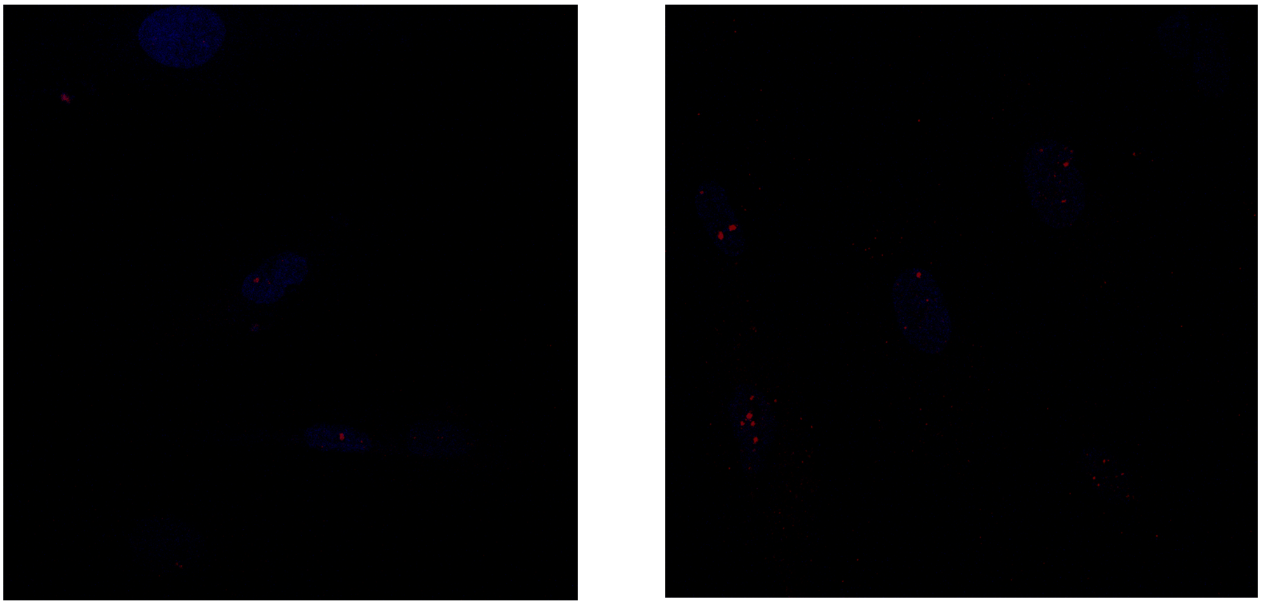

Figure S3. Confocal microscopy images of repair protein γ-H2AX in the nucleus of CAFs. Left: irradiated cells. Right: irradiated cells with GNP. Red dots correspond to DNA DSB damages and the blue stains are the cell nuclei.

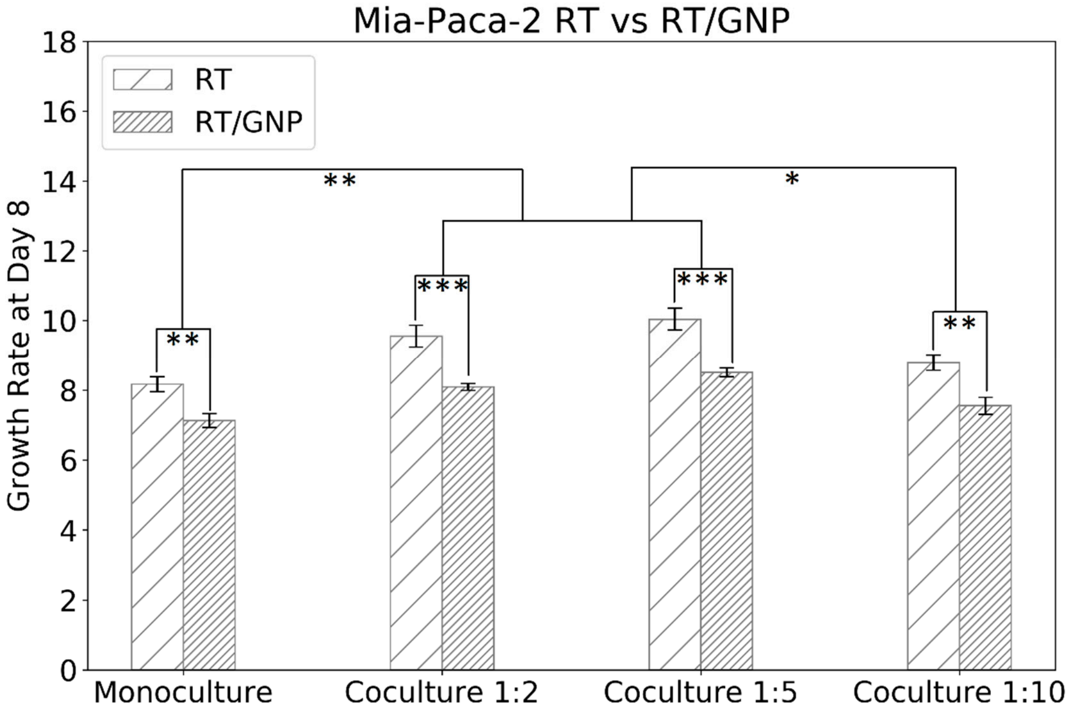

Figure S4. Mia-Paca-2 proliferation RT vs RT/GNP at the end of the experiment for monoculture vs the 3 different co-cultures. * indicates $p < 0.05$, ** indicates $p < 0.01$, *** indicates $p < 0.001$.
